# Proteinuria is Associated with Urinary Loss of Cubilin and Vitamin D-Binding Protein in Patients with Preeclampsia

**DOI:** 10.1038/s41598-020-60924-4

**Published:** 2020-03-03

**Authors:** Maria Clara Albejante, Tânia Cristina Macedo Kunz, Matheus Feliciano Costa Ferreira, José Humberto Zago Ribeiro Júnior, Robson José de Almeida, Lucas dos Santos Bacigalupo, Luiz Henrique Gomes Matheus, Maria Aparecida Dalboni, Cleber Pinto Camacho, Humberto Dellê

**Affiliations:** Postgraduate Program in Medicine, Universidade Nove de Julho – UNINOVE, São Paulo, Brazil

**Keywords:** Diseases, Medical research, Kidney diseases

## Abstract

Women with preeclampsia (PE) form a vulnerable group for vitamin D3 deficiency. Reabsorption of vitamin D3 occurs in the proximal tubule after being endocytosed in combination with DBP (vitamin D binding protein) by the megalin/cubilin receptor. Because proteinuria promotes tubule injury and dysfunction, we hypothesized that the proteinuria present in PE could promote the loss of these components into the urine. Twenty preeclamptic patients and ten normal pregnant women with a gestational age greater than 20 weeks composed three groups: NC, normotensive control pregnant patients; PE, non-proteinuric preeclamptic patients; and PPE, preeclamptic patients with proteinuria. When proteinuria was absent, preeclampsia was diagnosed accordingly to the American College of Obstetricians and Gynecologists’ (ACOG) guideline. The presence of 24-hour proteinuria equal to or greater than 300 mg was considered to form the PPE group. Urinary cubilin, megalin, and DBP were measured by ELISA and normalized by urinary creatinine. Regarding gestational age, there was no difference between the groups. NC group had arterial pressure within normal values, whereas PE and PPE groups had a significant increase (p < 0.01). As expected, PPE group presented elevated ACR (p < 0.05), accompanied by large amounts of cubilin and DBP in the urine (p < 0.05 vs. NC and PE). No difference was found in urinary megalin. PPE patients showed more chance of shedding cubilin into the urine compared to non-proteinuric patients (odds ratio 12.7 (1.2–136.3). In conclusion, this study further tightens the relationship between PE and vitamin D_3_ deficiency, since proteinuria present in PE induces the loss of molecules responsible for renal tubular vitamin D_3_ reabsorption for subsequent activation. Combined with other factors, the proteinuria may intensify vitamin D_3_ deficiency in PE.

## Introduction

Preeclampsia (PE) is a multisystem disorder of pregnancy in which renal damage has a significant contribution. A set level of proteinuria has been used to predict high-risk groups and define treatment for PE. In terms of physiopathology, proteinuria emerges due to processes that disturb the glomerular filtration barrier. Besides the classical mechanisms of glomerular damage such as endotheliosis and loss of podocytes^1,2^, an evidence for tubular injury have also been reported. Markers of proximal tubule injury such as kidney injury molecule-1 (KIM-1), neutrophil gelatinase-associated lipocalin (NGAL), and retinol-binding protein (RBP) were higher in the urine of PE patients than in urine of healthy pregnancies^3^ In addition, urinary lysosomal enzyme excretion is elevated in patients with PE compared to a pregnant woman with chronic hypertension^4^. Burwick *et al*. demonstrated that pregnancies affected by severe PE have high amounts of KIM-1 in the urine, which was closely correlated with urinary complement activation products, suggesting that tubular injury in PE is intensified by inflammation-activated complement^5^. Direct effects of proteinuria on tubular cell damage have been demonstrated. Proteinuria is associated with tubular atrophy and albumin-induced apoptosis^6^.

Proximal tubular dysfunction may reflect in disturbance of the vitamin D metabolism since the activation of 25-hydroxyvitamin D_3_ (25(OH)D_3_) in 1, 25 dihydroxyvitamin D_3_ (1,25(OH)D_3_) occurs in the proximal cells. 25(OH)D_3_ is a steroid; thus, it is complexed to vitamin D-binding protein (DBP) to circulate in the plasma. Because this complex is filtered in the glomeruli, a receptor-mediated uptake is required to prevent loss of 25(OH)D_3_ in the urine. For this reason, the brush border of the proximal tubule is equipped with megalin and cubilin, multiligand endocytic receptors responsibly by uptake of the filtered protein, including 25(OH)D_3_-associated DBP^7^. An endocytic vesicle is formed, and it´s fusion with lysosomes releases 25(OH)D_3_ to be converted to 1,25(OH)D_3_ by parathormone-regulated 1-α-hydroxylase.

Proteinuria-promoted tubular injury leading to a loss of endocytic apparatus components has been demonstrated. In nephrotic syndrome patients, tubular expression of megalin and cubilin was reduced^8^. Spontaneously hypertensive rats progressively loss megalin and cubilin into the urine^9^ and the treatment of hypertension preserves these molecules^10^.

Although PE patients present tubular injury induced by overload-protein in the tubular lumen, the consequence on the components of the endocytic apparatus was not yet demonstrated in this population. We hypothesized that the proteinuria present in PE could promote the loss of these components into the urine. In our study, we compared proteinuric PE patients with non-proteinuric patients and shown that proteinuria is critical for shedding cubilin and DBP into the urine.

## Methods

Thirty pregnancy were selected from a tertiary State Hospital of Sao Paulo city. The patients were distributed among three groups: NC, ten normotensive control pregnant patients; PE, fifteen non-proteinuric preeclamptic patients; and PPE, five proteinuric preeclamptic patients. When proteinuria was absent, preeclampsia was diagnosed if hypertension (≥140 mmHg systolic or ≥90 mmHg diastolic after 20 weeks gestation) occurred with markers of thrombocytopenia, impaired liver function, pulmonary edema or cerebral or visual disturbances, in accordance to the literature^11,12^. New-onset renal insufficiency was not considered as a criterion to avoid bias in the study. Thus, proteinúria was the main criteria to form the groups. Were considered positive for proteinuria women presenting total urinary protein ≥300 mg/24 h. The urine analysis was performed in the same hospital. Information such as maternal comorbidity, body, mass index, gestational age, and arterial pressure was also collected.

Inclusion and exclusion criteria were established to acquire a homogenous and free of non-related conditions group. Were included pregnant women regardless of socioeconomic level, older than 18 years, with a single gestation and living fetus with gestational age greater than 20 weeks, nulliparity or multiparous, reliable gestational age (date of last menstruation concordant with ultrasound up to 12 weeks gestation or at least two compatible ultrasound up to 20 weeks). We included patients that had not taken vitamin D replacement during prenatal care. The exclusion criteria were also applied under the following conditions: diabetes *mellitus*, chronic hypertension, renal diseases, systemic lupus erythematosus or antiphospholipid syndrome, use of legal/illegal drugs, presence of fetal malformations, severe clinical and/or obstetric complications. The protocol was approved by the Institutional Review Board (IRB) of the University Nove de Julho under the number IRB: 703.470.

Whole blood and urine were collected at the time of the emergency care (for preeclamptic patients) or in routine outpatient care (for NC women). The blood was collected in a tube containing EDTA and then was centrifuged for plasma collection, which was frozen at −80 °C protected from light. Urine samples were collected via a clean-catch specimen and centrifuged at −4 °C, with supernatant aliquoted and stored at −80 °C.

All procedures were performed in accordance with the relevant guidelines and regulations of the Institutional Review Board. All subjects gave written informed consent.

### Biochemical analysis

The spot urinary protein to creatinine ratio (PCR) and the albumin to creatinine ratio (ACR) were assessed. Serum creatinine was measured by the automated Jaffé method (CREA-Hitachi 912, Roche Diagnostics), with a sensibility of 0.04 ± 0.001 mg/dL. Albuminuria was determined by the automated immunoturbidimetric method (COBAS 6000, Roche Diagnostics), which has a sensibility of 0.0135 ± 0.0088 mg/dL.

Measurement of 25(OH)D_3_ and parathormone (PTH) was performed by electrochemiluminescence (ref 05894913 190 e 11972103 122, respectively, Roche Diagnostics, Mannheim, Germany), in an automated method (Cobas®, Roche Diagnostics, Mannheim, Germany). 25(OH)D_3_ > 75 nmol/L (30 ng/mL) was considered normal. PTH is presented in pmol/L, with normal values in a range of 1.6-6.8 pmol/L. All measurements were performed according to the company instruction.

### Measurement of cubilin, megalin, and DBP

Urinary concentration of cubilin (E-EL-H2463), megalin (E-EL-H2627), and DBP (E-EL-H2162) were measured using human quantitative enzyme-linked immunosorbent assay (ELISA), according to the protocol described by the manufacturer (Elabscience Biotechnology Co., Ltd,Hubei Province, China). One day before the assays, the samples were analyzed on 10% acrylamide gel for evaluation of protein integrity. Like performed to proteinuria and albuminuria, cubilin, megalin, and DBP were normalized by urinary creatinine.

### Statistical analysis

The data were presented as median with the maximum and minimum or mean and standard deviation. Kolmogorov-Smirnov test was used to verify sample distribution. ANOVA with post hoc Tukey method was used for analysis between groups. Correlation analysis was performed (Pearson or Spearman). The analysis of nominal or categorized variables was performed using the chi-square test. To categorize a continuous or ordinal variable, we used the best cut-off point established using an ROC curve and the Youden’s index. A p < 0,05 was considered significant. IBM SPSS Statistics for Windows, Version 22.0 from IBM Corp. Armonk, NY, USA, was used for statistical analysis and graphing.

## Results

The demographic characteristics are presented in Table 1. The sample consisted of Caucasian, brown and black women, with no statistical differences in racial composition between groups. Women were similar in age between the NC and PPE groups, and women in the PE group were older. None pregnant patients presented prenatal comorbidity. Regarding gestational age, there was no difference between groups. The sample consisted of Caucasian, brown and black women, with no statistical differences in racial composition between groups. Women were similar in age between the NC and PPE groups, and women in the PE group were older. None pregnant patients presented prenatal comorbidity.

**Table 1:** Demographic characteristics.

Variable	NC (n = 10)	PE (n = 15)	PPE (n = 5)	*P* Value
Maternal age, years [median (min-max)]	25 (20–31)	31 (23–38)	26 (18–33)	0.029
BMI 8–12 weeks (mean ± SD)	28.7 ± 4.9	33.4 ± 7.0	31.5 ± 5.1	0.222
Gestational age, weeks [median (min-max)]	36 (34–39)	38 (20–40)	34 (32–39)	0.769
SBP, mmHg (mean ± SD)	113.0 ± 8.2	149.4 ± 11.6	144.0 ± 10.9	0.007
DBP, mmHg (mean ± SD)	73.0 ± 8.2	103.9 ± 7.5	89.0 ± 9.4	<0.001

NC, normotensive controls; PE, preeclampsia; PPE, preeclampsia with proteinuria; min, minimum; max, maximum; BMI, body mass index; SBP, systolic blood pressure; DBP, diastolic blood pressure.

Regarding blood pressure, the NC group had systolic and diastolic pressures within normal values, whereas PE and PPE groups had significantly higher systolic and diastolic pressures (p < 0.01). There was no significant difference in blood pressure between the PE and PPE groups.

### Proteinuria and albuminuria

PCR was the criteria adopted to form the groups. Thus, the PCR of the PPE group was significantly higher compared to the other two groups (medians of 80.5 in the NC group, 108.0 in the PE group, and 790.3 mg/g in the PPE group; p < 0.05). Similarly, the ACR was significantly higher in the PPE group (517.6 mg/g) compared to NC and PE groups (medians of 8.7 and 30.5 mg/g, respectively; p < 0.05). A positive correlation was found between PCR and ACR (r = 0.90, p < 0.05).

### 25(OH)D_3_ and PTH

Serum 25(OH)D_3_ was measured and no difference was observed between the groups [median of 50.8 nmol/L (25.3–102.6) in the NC, 46.0 nmol/L (37.4–102.6) in the PE, and 49.3 nmol/L (32.7–66.7) in the PPE]. In this population, approximately 50% presented normal values for 25(OH)D_3_ (>50 nmol/L) [50% in the NC, 43% in the PE, and 50% in the PPE; p > 0.05]. Regarding PTH, the serum levels were not different between the groups [median of 2.4 pmol/L (1.8–5.4) in the NC, 3.6 pmol/L (1.9–5.4) in the PE, and 2.7 pmol/L (1.6–5.5) in the PPE].

### Cubilin, megalin and DBP

**Figure 1:**
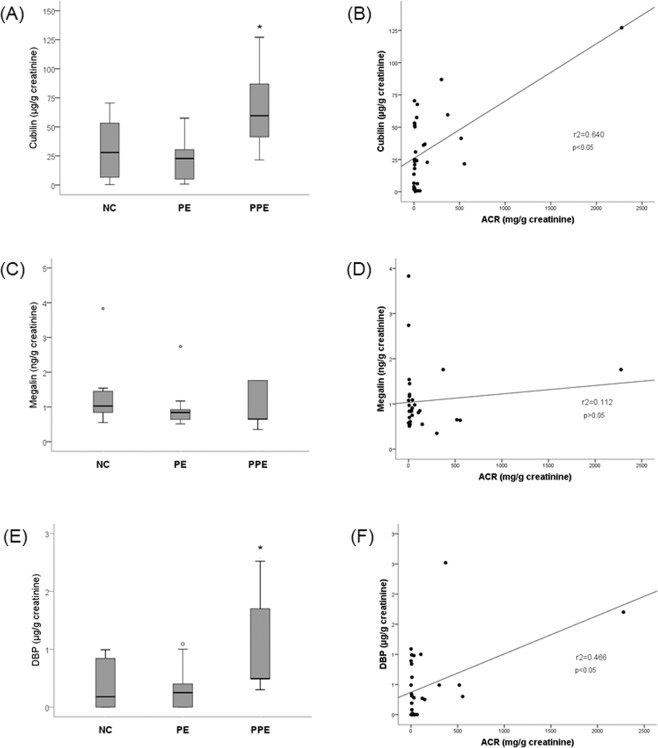
Boxplot graph of urinary concentration of cubilin (**A**), megalin (**C**) and DBP (**E**) in the three different groups (*p < 0.05 vs. NC and PE). Cubilin and DBP correlated positively to albumin-creatinine ratio (ACR) (1**B** and 1**F**, Pearson’s correlation). Urinary megalin was not significant correlated to albumin excretion (Spearman’s correlation).

Healthy pregnant presented basal levels of urinary cubilin, similarly to PE patients (32.1 ± 26.7 µg/g in the NC versus 22.0 ± 17.9 µg/g in the PE; p > 0.05). However, the shedding of cubilin into the urine was significantly increased in PPE women compared to NC and PE women (67.3 ± 41.1 µg/g in the PPE; p < 0.05 vs. NC and PE) (Fig. 1A). Megalin was found in the urine in small quantities and was not altered in PE or PPE women [median of 1.0 ng/g (0.6–3.8) in the NC, 0.8 ng/g (0.5–2.7) in the PE, and 0.6 ng/g (0.3–1.8) in the PPE]. Urinary amounts of cubilin and DBP correlated positively to albuminuria (Fig. 1B,F).

DBP was detected in the urine of NC and PE women, being significantly increased in PPE patients (0.30 ± 0.48 µg/g in the NC, 0.20 ± 0.41 µg/g in the PE, and 0.40 ± 0.55 µg/g in the PPE; p < 0.05). Women who have lost large amounts of DPB into the urine had no lower values of serum 25(OH)D_3_, thus, no correlation was found between DBP and 25(OH)D_3_ by either Spearman or Qui-square tests.

Using a ROC curve-derived cut-off for the different urinary molecules, was possible to identify higher population shedding large amounts of cubilin and DBP in the PPE group (p < 0.05). The chance of a PPE patient to have significant loss of urine cubilin is high (odds ratio 12.7 (1.2–136.3).

## Discussion

The loss of the tubular endocytic proteins responsible by 25-(OH)D intake had not been yet demonstrated in PE population. Our results demonstrate that proteinuria is associated with urinary loss of cubilin and DBP in preeclamptic patients.

The reasons by which the overload protein into the tubular lumen lead to tubule damage have been investigated for years. Glomerular ultrafiltration of massive amounts of plasma-derived proteins activates tubule cell apoptosis, tubule autophagy, phenotypic changes in proximal cells, mechanisms of tubulointerstitial inflammation and fibrosis, and complement leading to the tubular plasma membrane breakdown^13^. Megalin and cubilin have been described as localized in the apical membrane of the proximal tubular epithelial cells. Therefore, finding these molecules into the urine revels proximal tubular injury in experimental and clinical conditions. The loss of megalin and cubilin into the urine was demonstrated in diseases characterized by proteinuria. Fatah *et al*. demonstrated that immunostaining for megalin was significantly reduced in the proximal tubular epithelial cells of patients presenting with nephrotic syndrome due to minimal change disease, membranous nephropathy or focal segmental glomerulosclerosis (about half compared to controls)^8^. These results reinforce the Seki’s study, which demonstrated higher levels of urinary megalin in patients with membranous nephropathy correlating to the risk levels of requiring dialysis^14^. In order to prove that the loss of renal megalin immunostaining was promoted by proteinuria, Fatah *et al*. worked with the protein-overload proteinuria mice model. A threefold increase in urine protein/creatinine ratio was sufficient to reduce tubular megalin and cubilin immunostaining, as well as to increase urinary excretion of the same molecules on such animals^8^. The loss of cubilin and megalin was not only found in nephrotic syndrome, but also in situations of mild renal injury. The presence of microalbuminuria was associated with enhanced excretion of megalin and cubilin in type 1 diabetic patients^15^. Besides cubilin and megalin, high levels of DBP were found into the urine of type 1 diabetic patients presenting microalbuminuria^15^.

In our study, PPE patients presented a loss of cubilin into the urine, but not of megalin. Because cubilin has close interaction with megalin, it is expected that the megalin/cubilin complex may be pulled free from proximal tubular epithelial cells in the case of tubular damage. However, the megalin form released toward the tubular lumen depends on the severity of the tubular injury. Megalin undergoes regulated intramembrane proteolysis by which metalloproteinases catalyze ectodomain formation to be shed, while an intracellular domain is phosphorylated and maintained intracellularly^16^. Urinary excretion of the ectodomain of megalin has been associated with early diabetic nephropathy, whereas urinary full-length megalin has been linked to progressive chronic kidney disease in type 2 diabetic patients^17^. It is possible that in PPE patients only ectodomain of the megalin was released via urine and that the ELISA kit used targeted a region of the megalin arrested by the tubular wall, explaining the absence of difference between PPE and PE patients observed in our study.

PPE patients presented a significant loss of DBP into the urine, which was even positively correlated to urinary cubilin levels. In our point of view, this disturbance could potentiate predisposed pregnant to vitamin D deficiency. 25(OH)D_3_ was measured in the plasma, but no difference was observed between the groups. In addition, there was no correlation between serum concentrations of 25(OH)D_3_ and amounts of DBP into the urine. It is important to consider that about 50% of the pregnant patients of our study presented deficit of 25(OH)D_3_, even in NC women, which makes it hard to demonstrate the effect of the DBP shedding on serum 25(OH)D_3_. However, there are reasons to believe that isolated DBP shedding in urine is not sufficient to promote a decrease in serum 25-hidroxyvitamin D. In CKD patients, urinary loss of DBP is positively correlated to proteinuria, but is not associated with serum 25(OH)D_3_^18^. In addition, CKD patients show elevated levels of serum DBP, reinforcing the theory of a possible compensatory mechanism via hepatic production^18^. In type 2 diabetic patients, elevated amounts of urinary DBP were observed in micro- and macroalbuminuric patients, correlating positively to elevated levels of serum DBP^19^. Finally, interruption of proteinuria ameliorates the urinary loss of DBP but does not affect the vitamin D status in CKD patients^20^.

It is possible that the isolated loss of DBP in to the urine do not disturb vitamin D status, but in association with other factors may potentiate a preexistent deficit of vitamin D in preeclamptic patients. Further studies are necessary to confirm this hypothesis.

## Conclusion

Women with preeclampsia may have a disturbance of the tubule endocytic apparatus due to proteinuria resulting in shedding of DPB into the urine. The elucidation of this mechanism may increase the therapeutic repertoire against the complications caused by PE.

## References

[1] Stillman IE, Karumanchi SA (2007). The glomerular injury of preeclampsia. J. Am. Soc. Nephrol..

[2] Garovic VD (2007). Glomerular expression of nephrin and synaptopodin, but not podocin, is decreased in kidney sections from women with preeclampsia. Nephrol. Dial. Transplant..

[3] Xiao J, Niu J, Ye X, Yu Q, Gu Y (2013). Combined biomarkers evaluation fordiagnosing kidney injury in preeclampsia. Hypertens. Pregnancy..

[4] Torbé A (2014). Urinary lysosomal enzyme excretion in pregnant women with hypertensive disorders. Hypertens. Pregnancy..

[5] Burwick RM (2014). Complement activation and kidney injury molecule-1-associated proximal tubule injury in severe preeclampsia. Hypertension..

[6] Erkan E, Devarajan P, Schwartz GJ (2007). Mitochondria are the major targets in albumin-induced apoptosis in proximal tubule cells. J. Am. Soc. Nephrol..

[7] Nielsen R, Christensen EI, Birn H (2016). Megalin and cubilin in proximal tubule protein reabsorption: from experimental models to human disease. Kidney Int..

[8] Fatah H (2018). Reduced proximal tubular expression of protein endocytic receptors in proteinuria is associated with urinary receptor shedding. Nephrol. Dial. Transplant..

[9] Inoue BH (2013). Progression of microalbuminuria in SHR is associated with lower expression of critical components of the apical endocytic machinery in the renal proximal tubule. Am. J. Physiol. Renal Physiol..

[10] Arruda-Junior DF, Virgulino SG, Girardi AC (2014). Reduced tubular proteinuria in hypertensive rats treated with losartan is associated with higher renal cortical megalin expression. Horm. Mol. Biol. Clin. Investig..

[11] Tranquilli AL (2014). The classification, diagnosis and management of the hypertensive disorders of pregnancy: A revised statement from the ISSHP. Pregnancy Hypertens..

[12] Visintin C (2010). Guideline Development Group. Management of hypertensive disorders during pregnancy: summary of NICE guidance. B. M. J..

[13] Zoja C, Abbate M, Remuzzi G (2015). Progression of renal injury toward interstitial inflammation and glomerular sclerosis is dependent on abnormal protein filtration. Nephrol. Dial. Transplant..

[14] Seki T (2014). Significance of urinary full-length megalin in patients with IgA nephropathy. Plos. One..

[15] Thrailkill KM (2009). Microalbuminuria in type 1 diabetes is associated with enhanced excretion of the endocytic multiligand receptors megalin and cubilin. Diabetes Care..

[16] Zou Z (2004). Linking receptor-mediated endocytosis and cell signaling: evidence for regulated intramembrane proteolysis of megalin in proximal tubule. J. Biol. Chem..

[17] Ogasawara S (2012). Significance of urinary fulllength and ectodomain forms of megalin in patients with type 2 diabetes. Diabetes Care..

[18] Kalousova, M. *et al*. Vitamin D Binding Protein Is Not Involved in Vitamin D Deficiency in Patients with Chronic Kidney Disease. *Biomed. Res. Int*. 492365 (2015). 10.1155/2015/492365 PMC4434169 26064917

[19] Fawzy MS, Abu AlSel BT (2018). Assessment of Vitamin D-Binding Protein and Early Prediction of Nephropathy in Type 2 Saudi Diabetic Patients. J. Diabetes Res..

[20] Doorenbos CR (2012). Antiproteinuric treatment reduces urinary loss of vitamin D-binding protein but does not affect vitamin D status in patients with chronic kidney disease. J. Steroid. Biochem. Mol. Biol..

